# Pregnancy-Related Thromboembolism—Current Challenges at the Emergency Department

**DOI:** 10.3390/jpm14090926

**Published:** 2024-08-31

**Authors:** Ștefan-Ionuț Moroi, Emma Weiss, Silviu Stanciu, Elisabeta Bădilă, Adriana Mihaela Ilieșiu, Ana-Maria Balahura

**Affiliations:** 1 Department of Cardiology, Emergency Institute for Cardiovascular Diseases “Prof. Dr. C.C. Iliescu”, 022328 Bucharest, Romania; stefan-ionut.moroi@rez.umfcd.ro; 2 “Carol Davila” University of Medicine and Pharmacy, 050474 Bucharest, Romania; emma.weiss@umfcd.ro (E.W.); silviu.stanciu@umfcd.ro (S.S.); elisabeta.badila@umfcd.ro (E.B.); adriana.iliesiu@umfcd.ro (A.M.I.); 3 Internal Medicine Department, Bucharest Clinical Emergency Hospital, 014461 Bucharest, Romania; emma.weiss@umfcd.ro; 4 Dr. Carol Davila University Central Military Emergency Hospital, Calea Plevnei 134, 010825 Bucharest, Romania; silviu.stanciu@umfcd.ro; 5 Department of Cardiology, Colentina Hospital, 020125 Bucharest, Romania; elisabeta.badila@umfcd.ro; 6 Department of Cardiology, “Prof. Dr. Theodor Burghele” Clinical Hospital, 061344 Bucharest, Romania; adriana.iliesiu@umfcd.ro

**Keywords:** pregnancy, pulmonary embolism, emergency department

## Abstract

Thrombotic events during pregnancy are burdened by an increased risk of morbidity and mortality, despite innovations in their diagnosis and treatment. Given their multifactorial etiology, it is important to understand all the pathophysiological mechanisms but especially to achieve correct and timely diagnosis. Pulmonary embolism (PE) during pregnancy represents a rare event, with an incidence of 1 per 1000 pregnancies, but it is also one of the leading causes of death during pregnancy. Managing PE in the acute setting is even more challenging and complex due to the attempt to maintain a balance between hemorrhagic and thrombotic complications while ensuring an optimal outcome for both the mother and the baby. In this review, our aim is to analyze the most significant challenges of acute PE during pregnancy and identify suitable management approaches for specific situations in order to improve the prognosis of pregnant women.

## 1. Introduction

Venous thromboembolism (VTE) is an important cause of morbidity and mortality during pregnancy and the peripartum period, with an incidence of 1 per 1000 pregnancies [1]. Despite advances in the diagnosis and management of VTE, it remains a leading cause of maternal mortality worldwide. Pregnancy-related VTE can have both serious short- and long-term complications, including post-thrombotic syndrome, which can alter the quality of life of the mother [2].

Thromboembolic events are often generated by pregnancy-related risk factors, most of them being preventable [3]. Therefore, it is crucial to diagnose, prevent, and manage VTE, including its complications. However, this population comes with the physiological alterations associated with pregnancy and the potential risks that the imaging techniques present to the fetus, which makes an accurate diagnosis highly challenging. 

Therapeutic interventions must take into consideration the balance of the possible advantages and drawbacks of each approach to the mother and fetus [4]. In this regard, although it is a rare but deadly event, high-risk PE is the primary indication for thrombolysis, a challenging situation due to its high risk of bleeding complications.

However, data from a systematic review point out that thrombolysis in patients with high-risk PE has good maternal and fetal survival rates [5,6]. 

The aim of this review is to examine the key challenges of acute PE during pregnancy and to identify appropriate management strategies for different scenarios, with the goal of improving outcomes for pregnant women.

## 2. Epidemiology 

Pregnant and postpartum women have a six-time higher risk of VTE, compared with non-pregnant women. This risk also increases with gestational age. It is widely agreed that the greatest risk occurs during the postpartum period, with the incidence reaching its peak in the first six weeks after childbirth [6].

A meta-analysis that included 93 million pregnant and postpartum women revealed that the incidence of VTE was 1.2 per 1000 deliveries [7]. The global application of computer tomographic pulmonary angiography (CTPA) in pregnant and postpartum women has increased the rates of diagnosed PE during delivery admissions and during postpartum hospitalizations [8]. The incidence varies based on several risk factors and populations, with a higher incidence in women with a history of VTE or thrombophilia or those with diabetes or hypertension [9].

There are data that suggest racial differences in the occurrence of VTE during pregnancy and the postpartum period, with Black women being the most impacted. A potential explanation is the fact that Black women have a higher prevalence of certain risk factors, such as hypertension and sickle cell disease [10].

## 3. Pathophysiology of VTE in Pregnancy

Virchow’s triad, which consists of endothelial trauma, venous stasis, and hypercoagulability, all of which are increased during pregnancy, contributes to a higher risk of thrombus formation. These alterations in the coagulation and fibrinolytic systems are intended to minimize intrapartum blood loss but also increase the risk of thromboembolism [9]. Venous stasis of the lower limbs occurs due to changes in venous capacitance related to pregnancy and compression of the inferior vena cava or iliac veins by the gravid uterus. This augmentation of stasis in the lower limb venous system can occur even before the uterus has a marked increase in size [11]. There are also immunological changes, with a sudden increase in cytokines and vascular endothelial dysfunction that can lead to VTE [12]. 

Pregnancy and the postpartum period are also associated with a hypercoagulable state, with a physiologic increase in certain coagulation factors, such as factors II, VII, VIII, IX, and X. Conversely, there is also a reduction in the production of protein S and a decrease in the activation of the fibrinolysis inhibitors PAI-I and PAI-2 [13].

## 4. Risk Factors

Pregnancy itself is an important risk factor for VTE, but apart from it, other risk factors for thromboembolic events can be classified into maternal-specific, obstetric-specific, and new onset and transient risk factors [6]. Of note, traditional pre-existing risk factors are less prevalent in pregnant women compared with non-pregnant women who present with VTE [14].

Maternal-specific risk factors for venous thrombotic events in pregnancy include previous VTE, acquired or heritable thrombophilia, extensive varicose veins, body mass index > 30 kg/m^2^, maternal age > 35 years, parity of more than 2, smoking, pre-existing comorbidities, such as hypertension, gestational diabetes, heart failure, inflammatory bowel disease [15], or cancer [11]. Women who present with a history of both provoked and unprovoked VTE or with first-degree relatives diagnosed with inherited thrombophilia should be evaluated with a thrombophilia panel, including tests for antiphospholipid syndrome, factor V Leiden, and the prothrombin G20210A gene variant, as well as for deficiencies in antithrombin III, protein C, and protein S [15]. 

Obstetric-specific risk factors are current pre-eclampsia, multiple pregnancy, prolonged labor (>24 h), preterm birth, stillbirth, postpartum hemorrhage (blood loss exceeding 1 L or blood loss that requires transfusion), and Caesarean section [16]. 

Newly acquired or transient risk factors, which can be potentially reversible, may emerge later in pregnancy than when the initial risk assessment was conducted. Therefore, it is of utmost importance to carefully monitor the individual risk. These risk factors encompass hyperemesis gravidorum, ovarian hyperstimulation syndrome, fertility treatments including in vitro fertilization, postpartum sterilization, hospital admission or immobility, bone fracture, any surgical procedure during pregnancy or the postpartum period, existing systemic illness, and long distance travel exceeding 4 h [11].

## 5. Risk Assessment of Pregnancy-Related VTE

Considering that VTE is a leading cause of mortality, it is essential to assess the risk of developing VTE in all pregnant women as early as possible. Most women may exhibit one or more thrombotic risk factors during pregnancy. Conducting an initial risk assessment and implementing preventive strategies are essential in reducing the incidence of VTE in pregnant women [11]. Special attention should be given to women with a previous history of VTE, those with known thrombophilia, and those with a family history of VTE [17]. Those identified as having a high risk for VTE should receive both an antepartum and postpartum prophylactic or intermediate dose of low-molecular-weight heparin (LMWH) or unfractioned heparin (UFH) [18].

The risk scoring approach should evaluate the necessity of thromboprophylaxis during pregnancy and the postpartum period. Based on the associated risk factors, all women will be categorized into various risk levels, and the appropriate thromboprophylaxis regimen will be chosen. This approach can include observation alone, mechanical methods or LMWH, and the choice should be tailored based on each patient’s specific risk factors [19]. A scoring system to assess the risk of VTE in pregnant women would be highly beneficial, but unfortunately, no validated score currently exists for this purpose.

If pharmacological thromboprophylaxis is chosen as the appropriate regimen during pregnancy and the postpartum period, the risk of VTE must outweigh the risk of severe bleeding complications due to anticoagulation treatment. Recent guidelines and expert panels have taken this into consideration and suggested that pharmacologic prophylaxis should be considered only if the absolute risk of VTE exceeds 1 to 5%, with the prioritization of the woman’s preferences [20].

## 6. Clinical Presentation 

Clinical suspicion of deep vein thrombosis (DVT) is essential to ensure optimal diagnosis and treatment, because, without treatment, it may advance to PE, which can be life-threatening for both the mother and the fetus. DVT is significantly more prevalent in pregnant women compared to those who are not pregnant. In contrast to the general population, where thrombosis typically starts in the distal veins of the calf and progresses proximally, in pregnant women, it often originates in the proximal veins of the lower limb (primarily from iliac or femoral veins) due to compression by the pregnant uterus [4]. 

VTE during pregnancy can manifest as various forms, ranging from asymptomatic cases to severe and life-threatening conditions, such as hemodynamic unstable PE. Symptoms that suggest VTE in non-pregnant women include tachycardia, dyspnea, tachypnea, and lower limb edema and pain. However, these symptoms are non-specific and frequently encountered in various conditions, often imitating standard symptoms of pregnancy [9]. 

For DVT during pregnancy, the most frequent symptoms are pain and predominantly unilateral edema. These symptoms can be associated with warmth, erythema, or lower limb tenderness. Almost 80% of pregnant women with DVT exhibit these symptoms, yet the diagnosis is often overlooked [21]. In symptomatic pregnant women, DVT is more commonly diagnosed in the left lower limb, involving the proximal and iliac veins (70 to 90%) [22]. A possible explanation for this phenomenon is the dual compression of the left iliac vein, first by the normal anatomical crossing of the right iliac artery above the left iliac vein and second by the compression of the gravid uterus [4]. Although there are several scoring systems to evaluate the pre-test probability of VTE in non-pregnant women (such as Well’s criteria and the modified Geneva score), it is important to recognize that the studies validating these scoring systems did not include pregnant or postpartum women; therefore, their applicability to this population may be limited [4]. Chan et al. proposed a clinical prediction tool to aid in the diagnosis of DVT in pregnant women during the first trimester, which includes the following: (1) left lower limb symptoms; (2) more than 2 cm difference in calf circumference; (3) presentation during the first trimester of pregnancy. These three components are together referred to as the LEFt rule. If none of these factors is identified, the negative predictive value is 100%. The LEFt rule should, however, not be used as the only test to rule out DVT in pregnancy but rather as one component of a diagnostic approach, including other tests, such as D-dimer measurements and lower extremity duplex ultrasound (DUS) [23,24]. 

D-dimer levels increase during pregnancy compared with non-pregnant women, limiting the diagnostic value when evaluating the probability of VTE. However, normal D-dimer levels are a low-cost, non-invasive, simple test to rule out VTE during pregnancy in cases with low-to-intermediate clinical pretest probability [25]; conversely, a high D-dimer level necessitates an additional investigation, similar to the approach used for non-pregnant women [26]. Being widely available and without the risk of radiation to the fetus, DUS is the first-line diagnostic tool for symptomatic DVT. If DVT is found via DUS and PE is clinically suspected, additional chest imaging is not required, since the management approach would remain the same. Nevertheless, if PE is suspected clinically and DUS does not reveal DVT, a CT pulmonary angiogram (CTPA) or lung perfusion scintigraphy (V/Q scan) is required [27]. The detection of DVT in iliofemoral veins via compression ultrasonography (CUS) is often inadequate, due to the veins’ incompressibility from pregnancy-related changes in blood flow mechanics, their increased compressibility in the proximal veins, and their location within the pelvis. In this setting, adjunctive DUS parameters should be used, such as the venous flow changes with respiration and with the Valsalva maneuver in order to identify downstream occlusive thrombosis [28]. In pregnant women who are suspected of having iliac vein thrombosis, the diagnostic approach can be continued via magnetic resonance imaging (MRI) venography if the initial compressive ultrasound is negative. Also, if the CUS is negative but the clinical suspicion of DVT is high, reassessment with highly sensitive D-dimer testing on days 3 and 7 and/or repeat CUS should be performed. In cases of suspected pelvic DVT, CT venography can be considered; however, MRI venography is a valid option with excellent diagnostic precision, without exposing the mother and fetus to radiation [29]. Protocols without gadolinium should be taken into consideration, as fetal exposure to high doses of gadolinium is associated with developmental anomalies in small animals models [30], while evidence in humans is very limited [31]. It should be noted that MRI venography for DVT diagnosis is infrequent due to its restricted accessibility at the point of care.

The most frequent symptoms of PE, which are shortness of breath, tachycardia, and chest pain, are nonspecific and may also occur as normal physiological alterations during pregnancy, making the diagnostic management particularly challenging [32]. Varrias et al. demonstrated that sinus tachycardia is primarily regarded as a normal physiological aspect of pregnancy, but it is linked to adverse outcomes [33]. Therefore, a thorough assessment of the medical history, physical examination, laboratory evaluation, and diagnostic imaging are essential for the precise and prompt diagnosis of PE in pregnant women. The PERC (PE rule-out criteria) rule, used in the overall population to exclude PE in cases with a low pre-test probability, is insufficient during pregnancy [34]. Neglecting or missing a PE diagnosis can be fatal for both the mother and the child, whereas the careless use of imaging tests can expose both the mother and fetus to radiation [35]. A few rules of pre-test probability assessment have been proposed for pregnant patients with a working diagnosis of PE (Table 1). 

YEARS is an algorithm that can help in the treatment decision, which uses three clinical parameters: (1) clinical signs of DVT; (2) hemoptysis; (3) PE being the most probable diagnosis, in conjunction with the D-dimer levels. With this algorithm, PE can be excluded if all three criteria are absent and the D-dimer level is less than 1000 ng/mL, or if one or more of the three elements are present and the D-dimer level is <500 ng/mL. If PE cannot be ruled out by the YEARS criteria, further investigations need to be pursued [40]. Pregnant women with suspected PE and criteria for hemodynamic instability face a high risk of mortality in the initial hours and days. Therefore, it is recommended to start heparin anticoagulation without delay in those cases with a high or intermediate clinical probability of PE, even prior to confirming the diagnosis, while diagnostic tests are ongoing [27]. 

## 7. Risk Stratification of Pregnancy-Associated Pulmonary Embolism 

Risk stratification is crucial to adjust the best possible treatment of acute PE. It should take into consideration the impact of the elevated right ventricular (RV) afterload on RV performance, because the main cause of mortality in acute PE is circulatory failure [41].

The current European guidelines propose a two-step risk assessment approach [27]. High-risk PE is defined by the presence of hemodynamic instability or cardiac arrest, where reperfusion therapy is of utmost importance (systemic thrombolysis) because of the significant risk of mortality in the acute phase. Current criteria, aside from cardiac arrest, include obstructive shock with associated inadequate end-organ perfusion or persistent hypotension, defined as a systolic blood pressure (BP) < 90 mmHg or a fall in the systolic BP ≥ 40 mmHg without an alternative etiology. Intermediate-risk PE identifies hemodynamically stable patients, in which the presence of RV dilatation, increased troponin or increased B-type natriuretic peptide (BNP), or N-terminal-proBNP (NTproBNP) should emphasize careful hemodynamic monitoring due to the enhanced risk of short-term clinical deterioration. The reminder of PE patients are classified as low risk.

There is no research study that proves that this risk stratification applies to pregnancy-associated PE, but indirect evidence encourages that the physiological changes in pregnancy should not alter the current clinical assessment approach of hemodynamic compromise employed to identify high-risk PE [41].

Cardiac output increases by 50% during pregnancy to satisfy the metabolic demands of both the mother and the fetus. The heart rate rises, cardiac preload is elevated based on the increased blood volume, and cardiac afterload is decreased through a drop in systemic vascular resistance. This hyperdynamic state is associated with left ventricular eccentric remodeling [42]. Shortly after delivery, the hemodynamic parameters revert to pre-pregnancy levels. Because of this, the 90 mmHg threshold used to define high-risk PE is suitable in the context of pregnancy [41].

The biomarker criteria that indicate an intermediate risk of PE can also be applied during pregnancy. Troponin and NT proBNP fluctuate mildly during pregnancy and the postpartum period, and the 95th percentile stays under the non-gravid threshold levels of 14 ng/L and 300 ng/L, respectively [43]. Concerning RV dilatation, all heart chambers enlarge during pregnancy, including right heart dimensions [42]. Because the change in volume in left and right cavities is similar, it is not expected that the physiological RV/LV ratio will be altered in pregnancy [41].

Fetal distress can also indicate end-organ hypoperfusion. It is therefore proposed that, in late pregnancy, an obstetrical assessment of the fetal status using ultrasound and cardiotocogram should be performed, for the risk stratification of severe pregnancy-associated PE [41].

## 8. Diagnosis

Ongoing discussions in clinical practice focus on determining the most appropriate imaging modality for ruling out or diagnosing PE during pregnancy. The challenges associated with various modalities will be briefly discussed to aid in clinical decision-making [44].

A chest X-ray should be conducted to exclude other conditions, such as pneumonia or pneumothorax, that present with similar symptoms to those of PE. It should be noted that in up to 50% of cases, the thoracic X-ray can appear normal. The most frequent radiological findings in PE are pleural fluid accumulation, pulmonary edema, basal atelectasis, and localized opacities [45]. 

The electrocardiogram may suggest and strengthen the diagnosis of PE, with changes, such as sinus tachycardia, right bundle branch block, rightward axis deviation, or the S1Q3T3 pattern. It is important to highlight that these changes may not be present even in cases of a massive embolism [46].

Lung ultrasound (LUS) could potentially help in excluding or confirming some of the alternative diagnoses of PE, such as pleural effusion, pneumothorax, or interstitial syndrome, including pulmonary edema [47]. Moreover, the A profile in the BLUE protocol for LUS in conjunction with a positive DUS scan has 81% sensitivity, 99% specificity, and 98% negative predictive value for PE in non-pregnant patients [48]. Currently, there are no available protocols for LUS in pregnancy except for COVID-19 disease management [49]. However, a combination of POCUS (point of care ultrasound) with LUS, transthoracic echocardiography, and DUS could potentially improve the diagnostic accuracy, in addition to reducing the time to diagnosis of PE with a lower radiation-associated risk for the mother and fetus. 

In patients with hemodynamic instability, a transthoracic echocardiography (TTE) should be conducted, as it can rapidly detect acute RV dysfunction if acute PE is the cause of the hemodynamic deterioration [27]. When no imaging signs of RV dysfunction are identified, other causes of hemodynamic instability, such as acute coronary or aortic syndromes, pericardial tamponade, and acute valvular conditions, can be evaluated by TTE as well [32]. If PE is indirectly confirmed, all PE patients with hemodynamic compromise are considered for a rescue thrombolytic treatment, in the absence of contraindications. However, if a contraindication exists, alternative treatment options should be reviewed, such as percutaneous thrombectomy. Nonetheless, cardio-pulmonary resuscitation is necessary in patients with hemodynamic collapse and concomitant cardiac arrest, due to the very limited treatment options. Even though pregnancy is considered a relative contraindication for systemic thrombolytic treatment, current guidelines still suggest considering thrombolysis or surgical embolectomy as the primary reperfusion strategies in these patients [32]. A recent analysis of a contemporary cohort shows that 1 in 3 pregnant women with PE and hemodynamic instability underwent systemic thrombolysis [50].

**Figure 1:**
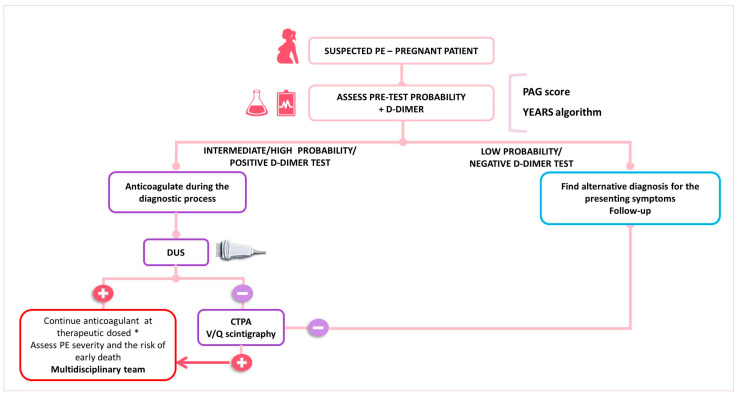
Proposed algorithm for the diagnosis of pulmonary embolism in pregnancy. CTPA—computer tomography pulmonary angiography, DUS—Doppler ultrasound, PAG—pregnancy-adapted Geneva score, V/Q—ventilation-perfusion; * See Table 2 and Table 3.

In women with suspected PE, the key components of the diagnostic algorithm are the assessment of pre-test clinical probability, combined with high-sensitivity D-dimer testing and bilateral lower limb CUS (Figure 1). If a high or intermediate pre-test probability exists, therapeutic anticoagulation should be started before waiting for the confirmation or ruling out the diagnosis [51]. In the presence of signs or symptoms of DVT, CUS needs to be carried out. If DVT is identified, the diagnosis of PE is indirectly confirmed. If proximal DVT is not identified or the CUS is not conclusive, thoracic X-ray and then ventilation/perfusion scintigraphy (V/Q scan) or CTPA are the recommended imaging modalities to rule out suspected PE. However, due to a low prevalence of confirmed PE in pregnant women (2 to 7%), several diagnostic challenges emerge, which translate in the different algorithms proposed for the confirmation of PE in pregnancy by the current guidelines [32,52]. Recently, a multicenter prospective study validated a diagnostic approach for pregnant women with suspected PE. This strategy combined a pre-test clinical probability assessment using the Geneva score, with high-sensitivity D-dimer tests, CTPA, and CUS (see Table 1). In cases with low or intermediate pre-test clinical probability combined with a negative D-dimer test result, PE was ruled out. All remaining patients underwent lower limb CUS; if this was negative, CTPA was performed. The study included 395 women, with PE confirmed in 28 (7.1%) and excluded in 367 (92.9%). Among untreated women with excluded PE, the rate of symptomatic VTE events was 0.0%. Thus, this diagnostic algorithm effectively rules out PE in pregnancy [26]. However, it is important to note that normal D-dimer values have been observed in patients with confirmed PE [53] further emphasizing the need for a systematic and individualized approach to the pregnant patient with suspected VTE.

The current ESC guidelines suggest that in pregnant women with suspected PE, an X-ray should be first performed. If the X-ray results are normal, a V/Q scan is recommended due to its low radiation exposure to both the fetus and the mother. If the X-ray reveals abnormalities, such as pulmonary opacities or infiltrates, a CTPA should be conducted directly [27]. Unfortunately, a V/Q scan at the point of care has a low availability, even more so outside working hours, which makes CTPA a frequent first choice as a diagnostic tool for PE suspicion [30].

## 9. Differential Diagnosis

The differential diagnosis of DVT in pregnant women is similar to that outside pregnancy. Several disorders presenting with warmth, erythema, edema, and pain in the lower limb, flank, pelvis, or back should be excluded. Such conditions can be superficial thrombophlebitis, cellulitis, lymphatic oedema, chronic venous disease, aneurysm of the popliteal vein or artery, Baker’s cyst, lymphadenopathy, hematoma, or muscle tears [11]. 

The clinical presentation of DVT in pregnancy can also resemble normal manifestations in pregnancy, such as cramps and lower limb swelling.

Symptoms of PE in pregnancy can range from mild dyspnea to severe shock. Other conditions that can mimic PE are pneumothorax, heart failure, peripartum cardiomyopathy, pneumonia, and acute aortic syndrome. It is crucial to recognize that PE can occur in conjunction with other disorders [11].

## 10. Treatment

### 10.1. Management of VTE in Pregnancy

The primary treatment for acute VTE during pregnancy and the post-partum period is anticoagulation (Table 2). The selection of the anticoagulant agent is determined by a few factors, including the anatomical location, extension and severity of the thrombosis, the gestational age, and the possible risks to the fetus. 

Heparin, preferably LMWH, is to be used, although UFH can be used as well, since neither agent crosses the placental barrier [54]. This differs from anti-vitamin K oral anticoagulant (AVK) warfarin that can cross the placenta and has the potential to cause severe complications, such as stillbirth, miscarriage, teratogenicity, pregnancy loss, neurodevelopmental deficits, and excessive bleeding. Nonetheless, warfarin can be used during breastfeeding. 

The direct oral anticoagulants (DOACs) pass through the placenta and are not recommended in pregnancy [54]. Moreover, LMWH is preferred as a first-line treatment for preventing and treating VTE in pregnancy instead of UFH because it exhibits a lower risk of adverse effects, such as hemorrhage, osteoporosis, heparin-induced thrombocytopenia and allergic reactions [55]. The initial dose of UFH is established according to the patient’s weight and then changed based on to the activated partial tromboplastin time (aPTT). Conversely, the dose of LMWH is based on the patient’s early pregnancy body weight (8–12 weeks of gestation) and administered twice daily, mostly without monitoring. However, monitoring the anti-activated coagulation factor X can be taken into account in patients with extreme body weights, significant renal impairment, or recurrent thromboembolic events [27]. 

Fondaparinux is another indirect factor Xa inhibitor, but its use during pregnancy is not routinely indicated as there is little evidence regarding efficacy and safety and a minor transplacental passage has been shown [56]. Therefore, it is not considered a first-line anticoagulant; however, it can be used with caution as an alternative choice for patients with heparin-induced thrombocytopenia [27]. 

The anticoagulant treatment duration in VTE and PE associated with pregnancy ranges from 3 to 6 months, including up to six weeks after delivery [9]. AVK and LMWH are recommended after delivery, while DOACs should neither be used during pregnancy nor during lactation. Extended anticoagulation is reserved for women with a history of VTE and two or more thrombophilias or for women with history of recurrent thrombotic events and any thrombophilia or women diagnosed with antiphospholipid antibody syndrome [57].

Several clinical situations require the careful assessment of risk versus benefit of anticoagulation for VTE in pregnancy, such as active bleeding or high-risk of bleeding: obstetrical risk factors, like placenta praevia, a low platelet count (bellow 75 × 109) or acquired coagulopathy, severe liver disease (with increased prothrombin time), a history of allergy, stroke in the past 28 days, or inadequately controlled hypertension (BP more than 200 mmHg systolic or 120 mmHg diastolic) [16]. 

Pregnant women with VTE need careful monitoring to evaluate their response to treatment, prevent recurrent VTE, and address potential complications. The nature and complexity of monitoring depends on a number of variables, including VTE severity, the type of anticoagulant administered, and the gestational age of the fetus [54]. Other important aspects to consider during follow-up are the coagulation parameters, as well as routine periodic fetal surveillance to evaluate fetal growth and welfare. 

Non-pharmacological treatment is also important in managing VTE symptoms in pregnancy. Compression stockings may assist in reducing the risk of post-thrombotic syndrome and alleviating symptoms, such as leg swelling or pain [58].

Additionally, pregnant women with VTE should be provided with information regarding the clinical aspects of recurrent VTE and be instructed to request medical assistance in the case of new or worsening symptoms [59]. 

### 10.2. Management of PE in Pregnancy

**Figure 2:**
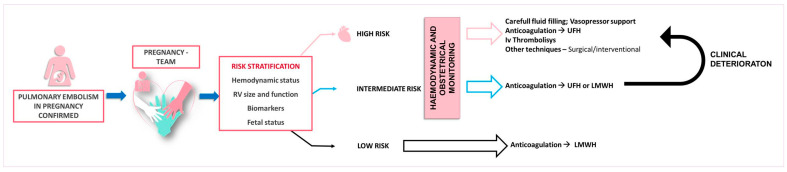
Proposed therapeutic management according to the pulmonary embolism severity. LMWH—low molecular weight heparin, UFH—unfractioned heparin.

The management of acute PE in pregnancy requires a comprehensive clinical assessment and risk evaluation, incorporating the hemodynamic status, RV function and size, imaging, biomarkers, and validated scoring systems for PE severity stratification [27]. A multidisciplinary approach consisting of cardiology, obstetrics, pulmonology, vascular medicine, hematology, anesthesiology/intensive care, cardiothoracic surgery, and interventional radiology is essential for decision making (Figure 2) [6].

Patients with acute low-risk PE, characterized by stable hemodynamics, normal RV function, and no end-organ damage, can be managed with LMWH or with UFH as an alternative option. These patients could potentially be managed on an outpatient basis [54].

Patients with hemodynamic stability but exhibiting RV strain on echocardiography or those with clinically severe PE—including oxygen saturation < 90%, tachycardia, tachypnea, numerous risk factors, or concurrent disease, such as old age, cancer, heart failure, or chronic pulmonary disease—are classified as intermediate risk. If troponin is elevated and RV dilatation/dysfunction is present, patients are additionally stratified into intermediate-high-risk groups as opposed to intermediate-low-risk groups in which troponin levels are normal. Sole anticoagulation is the mainstay treatment for intermediate-low-risk groups, while intensive monitoring is necessary for patients in intermediate-high-risk groups due to the potential for clinical worsening [27].

High-risk PE in pregnancy can be severe, with a case-fatality rate reaching as high as 37% [50]. If hemodynamic instability is present, UFH is administered as the primary treatment. Thrombolytic agents may be used if the hemodynamic status deteriorates. Immediate thrombolytic therapy is advised, provided there are no absolute contraindications to systemic thrombolysis [27]. 

Other treatment options of high-risk PE should be taken into account, such as surgical or percutaneous thrombectomy. If necessary, extracorporeal membrane oxygenation (ECMO) should be considered for depressurizing the RV and lung circulation [5]. Even though pregnancy is listed as a relative contraindication for thrombolysis, in women with circulatory collapse accompanied by cardiac arrest and the need for cardiopulmonary resuscitation, there are no alternative treatment possibilities [32]. Recent data demonstrated that as much as a third of high-risk PE women undergo systemic thrombolytic treatment. Nonetheless, thrombolysis seems to be associated with a favorable outcome, with 94% maternal survival and 88% fetal survival. Frequent side effects following thrombolytic treatment are bleeding complications, with a reported frequency of 18% during pregnancy up to 58% of cases in the post-partum period [5].

The peripartum period, along with spinal and epidural anesthesia, carries a high risk of bleeding; thus, thrombolysis should be performed peripartum only in a life-threatening situation [32]. Fibrinolytic drugs do not cross the placental barrier, so the fetal risk is low [60]. However, the lack of studies rule out conclusions regarding the safety profile and efficacy of thrombolysis in pregnancy high-risk PE. Therefore, important maternal or fetal adverse reactions cannot be extrapolated on for the use of a thrombolytic agent only [32].

If absolute contraindications are present, other treatment strategies need to be provided, such as percutaneous low-dose thrombolysis (CDT), surgical embolectomy, or thrombectomy [61]. Several small studies confirmed that CDT, in intermediate- and high-risk PE, is associated with better outcomes concerning bleeding complications compared to systemic thrombolysis [62]. In the post-partum period, surgical pulmonary embolectomy and percutaneous thrombectomy can be considered suitable treatment options to mitigate the hemorrhagic risks associated with systemic thrombolytic therapy. Nonetheless, these methods are usually not readily available and are used only in a life-threatening situation as a bailout therapeutic strategy [5].

In cases with hemodynamic instability where reperfusion therapy is not available or effective, recent data suggest that a bridging therapy with the transit use of mechanical circulatory support via ECMO can improve outcomes until mechanical thrombolysis or embolectomy is available [63]. ECMO has not been widely used in patients with acute high-risk PE and pregnancy. Limited date from a systematic review of 21 pregnant women with PE report a maternal survival rate of 76%, and a fetal survival rate of 63% with the use of ECMO [41]. Table 3 describes the current options for the treatment of high-risk PE with hemodynamic instability.

**Table 3:** Treatment for high-risk PE with hemodynamic instability. LMWH—low-molecular-weight heparin; UFH—unfractioned heparin; PE—pulmonary embolism.

Technique	Description
**Anticoagulation**	UFH is a first-line anticoagulation therapy in a patient with hemodynamic instability. Since they do not penetrate the placental barrier, they are safe during pregnancy and breastfeeding [53]. Can be given both intravenously or subcutaneously [44].
**Thrombolytic treatment**	Systemic thrombolysis is recommended only for high-risk patients with hemodynamic instability [27]. In patients with massive PE, favorable maternal outcomes were observed. The rate of maternal survival is approximatively 92% [5]. Lethal complications, including cardiac arrest or severe maternal hemorrhage, may arise [44]. Alteplase is given at a dose of 100 mg administered over two hours [41].
**Catheter-directed therapy (CDT)**	In high-risk patients where thrombolysis and anticoagulation have failed or are contraindicated, CDT can be beneficial [64]. The incidence of major hemorrhage is infrequent, about 18% [65]. The devices use mechanical fragmentation, aspiration, or thrombolytic infusion [44]. Should be conducted only in experienced centers [41].
**Surgical thrombectomy**	It is particularly taken into account for treating PE during pregnancy when anticoagulation is insufficient or the patient has hemodynamic instability [66]. Surgical thrombectomy with cardiopulmonary bypass is typically carried out without cardioplegia involving the removal of pulmonary clots through surgical openings in the two primary pulmonary arteries. Maternal survival occurred in about 86% of patients [5].
**Extracorporeal membrane oxygenation (ECMO)**	In patients with high-risk PE, it is regarded as a lifesaving intervention [67]. Venous and arterial cannulas are inserted into the inferior vena cava and the common femoral artery [44]. To improve outcomes, it can be combined with fibrinolysis or embolectomy [68]. The most suitable indication for ECMO is unresponsive cardiac arrest resulting from PE [44].

Inferior vena cava (IVC) filters can be taken into consideration in gravid women when an absolute contraindication to anticoagulation is present or when recurrent PE occurs despite optimal anticoagulant treatment in order to prevent further embolic events in the pulmonary circulation. However, data on this approach are limited. In the RIETE registry, recruiting between the years 2001 and 2019, a cohort of women with VTE, either pregnant or in the postpartum period, only one patient from the 34 receiving an IVC filter had a complication (vein tear during filter recovery) [14]. In a systematic review analyzing 124 pregnant women with DVT who underwent an IVC filter implantation, no fatal PE occurred after device placement, and the complication rates from retrieval were similar with those observed in the general population. The most common adverse events are migration, perforation, fracture, or death [69]. However, despite the authors’ conclusion that IVC filters can be used effectively in pregnant women to prevent PE, more studies are needed to suggest that they can be used routinely [70].

In general, the evidence supporting advanced therapeutic options in pregnant women with high-risk PE is lacking. Due to the complexity of diagnosis and treatment, optimal treatment strategies should be individualized and based on a multidisciplinary team approach with expertise in managing PE during pregnancy [27].

## 11. Conclusions

It is widely acknowledged that pregnancy involves physiological changes that increase the risk of thromboembolic events. VTE, as well as its complications, is an important cause of morbidity and mortality in the mother and the fetus. Hence, it is crucial to promptly and accurately identify women for whom preventive anticoagulation would be beneficial. PE is a rare event, but with a high-risk of mortality, especially during pregnancy. The diagnosis of acute PE in pregnant women can be difficult due to significant overlap between symptoms due to embolism and symptoms secondary to the inherent anatomical and physiological changes in pregnancy; a tailored diagnostic algorithm should be implemented based on currently available pre-test probability assessment tools along with D-dimer testing, DUS, CTPA, and V/Q scans. However, PE with hemodynamic instability is a major emergency, and this diagnosis needs to be established as soon as possible, utilizing the appropriate diagnostic tools in order to implement a prompt and immediate reperfusion strategy. Although pregnancy is regarded as a relative contraindication for thrombolysis, it should still be considered for pregnant women with high-risk PE, alongside other therapeutic approaches, including low-dose CDT, surgical embolectomy, or percutaneous thrombectomy. Overall, decision-making should be supported by a multidisciplinary approach, informed by guideline recommendations and currently available data, with careful consideration of the benefits and risks of the mother, as well as the fetus, available resources, and the level of expertise.

## Figures and Tables

**Table 1:** **Clinical pre-test assessment of DVT and PE**. CTPA—computer tomography pulmonary angiography, DVT—deep vein thrombosis, I—intermediate, L—low, LL-DUS—lower limb Doppler ultrasound, pts.—patients, PE—pulmonary embolism, V/Q scan—ventilation/perfusion scan, Sb—sensibility, Sp—specificity, VTE—venous thromboembolism.

Clinical Pre-Test Probability Assessment	Components and Interpretation	Efficiency	Studies Evaluating Pregnancy-Adapted Algorithms for the Diagnosis of PE Including the Specified PTP Scores
**LEFt rule** **(for DVT in pregnancy)** [24]	Left lower limb symptoms A difference in calf circumference exceeding 2 cm Presentation in the first trimester LEFt rule score interpretation If all factors are absent, the negative predictive value of DVT is 100%	Sb 100% Sp 50%	NA
**Revised Geneva score(for PE)** [36]	Age ≥ 65 years—1p Previous DVT or PE—3p Surgery or fracture within 1 month—2p Active cancer—2p Unilateral lower limb pain—3p Hemoptysis—2p Heart rate 75 to 94 bpm—3p Heart rate 95 or more bpm—5p Pain on deep palpation of the lower limb accompanied by unilateral swelling—4p Revised Geneva score interpretation Low probability of PE < 4p Intermediate probability of PE 4–10p High probability of PE > 10p	Sb 70% Sp 52%	**CT-PE pregnancy study** Multicenter, multinational, prospective **Algorithm**: - L or I pretest probability + negative D-dimer = rule-out PE otherwise LL-CUS - Negative LL-CUS, CTPA was performed. A V/Q scan was done if CTPA inconclusive. **Results**: Rate of symptomatic VTE 0.0% (95% CI, 0.0% to 1.0%) among untreated pts. [26]
**The Pregnancy-Adapted Geneva (PAG) score** **(for PE in pregnancy)** [37]	Age 40 years or over—1p Previous DVT or PE—3p Surgery or lower limb fracture within 1 month—2p Unilateral lower limb pain—3p Hemoptysis—2p Heart rate > 110 bpm—5p Pain on deep palpation of the lower limb accompanied by unilateral swelling—4p Revised Geneva score interpretation Low probability of PE 0–1 p Intermediate probability of PE 2–6 p High probability of PE ≥ 7p	Reported AUC	Needs further external validation
**YEARS**	YEARS Items Clinical signs of DVT Hemoptysis PE most likely diagnosis YEARS rule score interpretation 0 YEARS items and D-dimer < 1000 ng/mL − PE excluded 0 YEARS items and D-dimer ≥ 1000 ng/mL − Order CTPA ≥1 YEARS items and D-dimer < 500 ng/mL − PE excluded ≥1 YEARS items and D-dimer ≥ 500 ng/mL − Order CTPA	Sb 90% Sp 65%	**1. ARTEMIS** Prospective, multicenter, international study **Algorithm**: Adaptation of YEARS algorithm by including LL-DUS in pts with symptoms of DVT. - DVT present—patient received AC, no CTPA - DVT negative and all other pts. in which PE was not ruled out—underwent CTPA **Results**: From the ruled-out PE pts, 1 patient was diagnosed with VTE during follow-up (0.21%; 95% CI, 0.04 to 1.2) CTPA (thus radiation) was avoided in 32–65% of pts. [38] **2. The YEARS algorithm in the CT-PE population.** Post-hoc analysis of outcomes from a prospective study on PE diagnosis in pregnant women **Algorithm**: 371 pregnant patients from the original CT-PE study—retrospective application of the YEARS algorithm. **Results**: Failure rate of YEARS algorithm 0 out of 77 pts (95% CI, 0.0–3.9) [39]

**Table 2:** Anticoagulant options for VTE during pregnancy. APTT—activated partial thromboplastin time; DOAC—direct oral anticoagulant; INR—international normalized ratio; LMWH—low molecular weight heparin; UFH—unfractioned heparin; VKA—vitamin K antagonist; VTE—venous thromboembolism; kg—kilogram; i.v.—intravenous. ^&^ Based on the early pregnancy weight (8–12 weeks). * Fondaparinux can be used exceptionally in pregnancy in patients with heparin-induced thrombocytopenia [17,22,50].

Anticoagulant	Prophylactic Dose	Therapeutic Dose ^&^	Recommended during Pregnancy	Recommended during Breastfeeding	Crossing of the Placental Barrier
**UFH**	3 × 5000 Units/day 2 × 7500 Units/day	80 Units bolus i.v., followed by 18 Units/kg/h i.v. Target APTT: 1.5–2 × baseline	Yes	Yes	No
**LMWH**			Yes	Yes	No
- Dalteparin	1 × 5000 Units/day	1 × 200 Units/kg/day 2 × 100 Units/kg/day			
- Enoxaparin	1 × 4000 Units/day	2 × 100 Units/kg/day 1 × 150 Units/kg/day			
- Nadroparin	1 × 2850 Units/day	2 × 85 Units/kg/day			
- Tinzaparin	1 × 4500 Units/day	1 × 175 Units/kg/day			
**FONDAPARINUX**	1 × 2.5 mg/day	<50 kg: 1 × 5 mg/day 50–100 kg: 1 × 7.5 mg/day >100 kg: 1 × 10 mg/day	No *	Yes	Yes
**VKA**	-	INR between 2–3 Preferably <5 mg/day	No past 6 weeks gestation. First trimester embriopathy Can be taken into consideration in women with mechanical heart valves	Yes	Yes
**DOAC**	-	-	No	No	Yes

## Data Availability

The data presented in this study are available upon request from the corresponding author.
